# Targeting Haemagglutinin Antigen of Avian Influenza Virus to Chicken Immune Cell Receptors Dec205 and CD11c Induces Differential Immune-Potentiating Responses

**DOI:** 10.3390/vaccines9070784

**Published:** 2021-07-13

**Authors:** Angita Shrestha, Jean-Remy Sadeyen, Deimante Lukosaityte, Pengxiang Chang, Marielle Van Hulten, Munir Iqbal

**Affiliations:** 1 The Pirbright Institute, Ash Road, Pirbright, Woking, Surrey GU24 0NF, UK; angita.shrestha@pirbright.ac.uk (A.S.); jean-remy.sadeyen@pirbright.ac.uk (J.-R.S.); deimante.lukosaityte@pirbright.ac.uk (D.L.); pengxiang.chang@pirbright.ac.uk (P.C.); 2 Department of Zoology, Peter Medawar Building, South Parks Road, University of Oxford, Oxford OX1 3SY, UK; 3 Global Poultry R&D Biologicals Boxmeer, Intervet International BV, MSD Animal Health, Wim De Körverstraat 35, 5831 AN Boxmeer, The Netherlands; marielle.vanhulten@merck.com

**Keywords:** targeted antigen delivery vaccine, antigen presenting cells, single chain fragment variable antibodies, dendritic cell receptor for endocytosis-205, cluster of differentiation 11c, avian influenza virus, haemagglutinin

## Abstract

Improving the immunogenicity and protective efficacy of vaccines is critical to reducing disease impacts. One strategy used to enhance the immunogenicity of vaccines is the selective delivery of protective antigens to the antigen presenting cells (APCs). In this study, we have developed a targeted antigen delivery vaccine (TADV) system by recombinantly fusing the ectodomain of hemagglutinin (HA) antigen of H9N2 influenza A virus to single chain fragment variable (scFv) antibodies specific for the receptors expressed on chicken APCs; Dec205 and CD11c. Vaccination of chickens with TADV containing recombinant H9HA Foldon-Dec205 scFv or H9HA Foldon-CD11c scFv proteins elicited faster (as early as day 6 post primary vaccination) and higher anti-H9HA IgM and IgY, haemagglutination inhibition, and virus neutralisation antibodies compared to the untargeted H9HA protein. Comparatively, CD11c scFv conjugated H9HA protein showed higher immunogenic potency compared to Dec205 scFv conjugated H9HA protein. The higher immune potentiating ability of CD11c scFv was also reflected in ex-vivo chicken splenocyte stimulation assay, whereby H9HA Foldon-CD11c scFv induced higher levels of cytokines (IFNγ, IL6, IL1β, and IL4) compared to H9HA Foldon-Dec205 scFv. Overall, the results conclude that TADV could be a better alternative to the currently available inactivated virus vaccines.

## 1. Introduction

There has been a remarkable increase in the poultry production and trade in the past two decades [1]. However, the widespread of avian influenza virus (AIV), Newcastle disease virus (NDV), Marek’s disease virus (MDV), and infectious bursal disease virus (IBDV) is causing major threats to poultry production as well as posing a credible zoonotic and pandemic risks [2,3,4,5]. Vaccines are an important component of poultry disease prevention and control worldwide. However, many currently available poultry vaccines have several drawbacks including long production times, administration of multiple doses, difficulty in differentiating vaccinated from the infected animals (DIVA), and interference from maternal derived antibodies [6]. Therefore, there is a need to improve the efficacy of vaccines. Various strategies have been developed to enhance the immunogenicity of vaccines. One such strategy is the recombinant targeted antigen delivery vaccine (TADV) whereby protective antigens are selectively delivered to professional antigen presenting cells (APCs) such as dendritic cells (DC), macrophages and B cells [7]. Such antigen targeting to APCs can be carried out by conjugating antigens to either ligands of pattern recognition receptors—i.e., pathogen associated molecular patterns (PAMPs) like lipopolysaccharides (LPS) and CpG oligonucleotides or to antibodies specific for receptors on APCs. In antibody-based targeting, antigens are either chemically conjugated to monoclonal antibody (mAb) specific for selected APC receptors or genetically engineered where the antigen is fused to antibody fragments such as single chain fragment variable (scFv) or fragment antigen binding (Fab), specific for the APC receptors [8,9,10]. The scFv antibodies have been increasingly used for antigen targeting due to their small size. They are 25–30 kDa in size and are the smallest unit of immunoglobulin molecule that hold a complete antigen binding domain [11]. They consist of variable heavy (vH) and variable light (vL) chains, which are joined together by a flexible peptide linker (mostly glycine and serine stretches) [12]. They offer several advantages over whole antibody for antigen targeting. They lack Fc domain and hence, provide specific antigen binding affinity to APCs receptors by reducing non-specific uptake of antigen [13]. 

One of the most commonly used antigen targeting receptor in mammalian studies is DC receptor for endocytosis-205 (Dec205) [14,15,16]. Dec205 is a C-type lectin endocytic receptor of the mannose receptor family and is shown to enhance antigen presentation via the major histocompatibility complex II (MHC II) pathway [9]. Chicken Dec205 has about 51% and 48% amino acid sequence similarity to human and mouse Dec205, respectively [17]. In chickens, low-level expression of Dec205 has been detected in CD4^+^, CD8^+^, and γδ T lymphocytes; B lymphocytes; and macrophages with most expression on DCs [17]. The first-in-human study of a protein vaccine targeting APCs was conducted with CDX-1401 vaccine (Celldex Therapeutics Inc., New Haven, Connecticut, USA) which is an anti-cancer vaccine targeting human Dec205 receptor. This vaccine was proven to be safe and efficacious in the phase I clinical trials [18]. Similarly, the first antigen targeting study in chickens was also directed towards Dec205 receptor where a complete HA protein of H5N2 AIV was chemically conjugated to chicken Dec205 mAb. A single dose of this vaccine was shown to be sufficient to elicit a strong antibody response in chickens as early as 14 days after priming [16]. Other receptors used in mammals for antigen targeting include cluster of differentiation 11c (CD11c), CD40, Clec9A, and MHC II [19,20,21,22,23,24,25]. CD11c receptor is another important receptor of interest for antigen targeting studies in mammals. CD11c belongs to the family of Beta 2 (β2) integrins and is highly expressed on DCs, macrophages, natural killer cells (NK), and activated T cells [26]. CD11c antibodies have been used to selectively stain chicken DCs, NK cells, and macrophages [27,28]. Studies have shown that targeting antigens to CD11c can promote rapid and high antibody responses in mice [21,29,30,31]. It was demonstrated that 100 ng of albumin protein (OVA) conjugated to anti-CD11c Fab was able to generate anti-OVA antibody titres greater than those produced by a 100-fold higher dose of OVA in complete Freund’s adjuvant [21]. Hence, CD11c represents an efficient target for antigen targeting studies.

The strategy of antigen targeting has not been explored much in avian system for modulating the immunogenicity of poultry vaccines [16]. In this study, we used haemagglutinin (HA) protein of H9N2 AIV as a model antigen for targeting. The HA protein lacking transmembrane domain (TM) was fused to scFv antibodies specific for chicken Dec205 and CD11c receptors and produced as a soluble trimeric protein in *Drosophila melanogaster* S2 cells. The immunogenicity of Dec205 scFv and CD11c scFv targeted H9HA was compared in chickens. To the best of our knowledge, this is the first report of CD11c receptors being used in chickens for antigen targeting studies. 

## 2. Materials and Methods

### 2.1. Ethics Statement

All animal studies and procedures were carried out with the guidance and regulations of European and United Kingdom Home Office regulations under project licence number P68D44CF4. All animal work was approved by the Animal Welfare Ethical Review Board (AWERB) at The Pirbright Institute.

### 2.2. Viruses and Cells

A/Chicken/Pakistan/UDL 01/2008 (UDL 01/08) H9N2 virus was grown in 10-day-old specific pathogen free (SPF) embryonated hens’ eggs (VALO BioMedia, Osterholz-Scharmbeck, Germany), and obtained by harvesting the allantoic fluid by centrifugation at 3000 rpm for 20 min. The virus was titrated by plaque assay or TC1D_50_ (50% tissue culture infective dose) on Madin–Darby canine kidney (MDCK) cells. The inactivation of the virus was carried out using 0.1% beta-propiolactone (BPL, Alfa Aesar, Haverhill, MA, USA). Three blind passages were performed in 10-day-old SPF embryonated hens’ eggs to confirm inactivation. The inactivated virus was purified by ultracentrifugation through a continuous 30–60% *w*/*v* sucrose gradient.

### 2.3. Construction of scFv and H9HA Fused scFv Antibodies Expressing Plasmids

The hybridoma clone IAH F877:AD6 producing monoclonal antibodies recognising chicken Dec205 was produced in mice at the Pirbright institute (formerly known as Institute of Animal Health (IAH) [17]. Chicken Dec205 CTLD 4-5-6 (GenBank accession number: AJ574899) was used as immunogen for raising monoclonal antibodies against chicken Dec205 [17]. Hybridoma for putative chicken CD11c (clone: IAH 8F2) was gifted to the institute by Bernd Kaspers (University of Munich, unpublished). Both the hybridomas were sequenced commercially (Absolute Antibody Ltd., Oxford, UK). The gene sequences comprising the variable light (vL) and variable heavy (vH) chain of chicken Dec205 and CD11c mAb were joined by flexible peptide linker (Gly_4_Ser)_4_ to construct Dec205 scFv and CD11c scFv, respectively. The scFv antibody gene sequences were manufactured commercially (Geneart Life Technologies, Carlsbad, CA, USA) and cloned into *Drosophila melanogaster* expression vector (pMT-BIP-V5-His version A, Life Technologies) using the *Not I* and *Xba I* restriction sites (Figure S1a). The resultant vector pMT-BIP-scFv-V5-His was used to insert ectodomain of H9HA gene that lacks both HA gene signal peptide and the TM domain, replaced with a 30 amino acid trimerisation foldon sequence of the trimeric protein fibritin from bacteriophage T4 [32] (hereinafter referred to as rH9HA), using *Kpn I* and *Pac I* restriction sites (Figure S1b). The resultant vector was named pMT-BIP-rH9HA-scFv-V5-His. The HA protein used for making the recombinant subunit AIV vaccine was synthetically produced by incorporating consensus sequence of HA of H9N2 viruses derived from analysis of over 2000 H9HA sequences of G1-like H9 virus lineage using Minimum Sphere Consensus (MScon) method [33]. This synthetic HA has 98% amino acid sequence similarity to UDL 01/08 H9N2 virus HA ectodomain (GenBank accession number: ACP50708.1, HA1: 19-338 and HA2: 339-560).

### 2.4. Expression and Purification of scFv and H9HA Fused scFv Antibodies

All the recombinant proteins were expressed using Drosophila Expression System (DES^®^, Life technologies). The expression plasmids were transfected into *Drosophilla melanogaster* Schneider 2 (S2) cells using calcium phosphate transfection kit, according to manufacturer’s protocol (Life Technologies). Stable S2 transfectants were generated by adding hygromycin to a final concentration of 250 μg/mL every week for at least 4 weeks. Single cell clone expressing recombinant proteins was obtained via the limiting dilution method [34]. Recombinant proteins were secreted into culture supernatant after CuSO_4_ (500 µM) induction and then purified using Profinity™ IMAC uncharged column (Bio-Rad, Hercules, CA, USA). The purified proteins were analysed by using 10–12% sodium dodecyl sulphate–polyacrylamide gel electrophoresis (SDS-PAGE) followed by Coomassie blue (Life Technologies) staining. The concentration of the purified proteins was measured using Pierce BCA Protein Assay Kit (Life Technologies) according to the manufacturer’s protocol.

### 2.5. Characterisation of scFv and H9HA Fused scFv Antibodies

Enzyme linked immunosorbent sssay (ELISA) was carried out to examine if Dec205 scFv and rH9HA-Dec205 scFv can detect and bind to chicken Dec205 receptor protein. The coding sequence of chicken Dec205 C-type lectin domains (CTLD) 4-5-6 [17] was cloned into pMT-BIP-His vector for expression in *Drosophila melanogaster* S2 cells. Briefly, 8 μg of chicken Dec205 CTLD 4-5-6 was added onto the first well of 96 well maxisorp ELISA plates (Life Technologies), and a two-fold dilution was carried out in carbonate buffer (15 mM NaCO_3_, 35 mM NaHCO_3_, 3 mM NaN_3_). The plates were incubated at 4 °C overnight. For detection, the plates were incubated with either Dec205 mAb (1 μg/mL) or equimolar concentration of Dec205 scFv and rH9HA-Dec205 scFv for 1 h at 4 °C. This was followed by further incubation with horseradish peroxidase (HRP) conjugated goat anti-mouse secondary antibody (1:1000, Bio-Rad, primary antibody: Dec205 mAb) or anti-V5 HRP secondary antibody (1:1000, Bio-Rad, primary antibody: Dec205 scFv or rH9HA-Dec205 scFv). The colorimetric detection was carried out by adding tetramethylbenzidine (TMB) substrate (Life Technologies) and read at wavelength 450 nm in ELx808 absorbance microplate reader (BioTek). For the western blot analysis, chicken Dec205 CTLD 4-5-6 was run on a 10% SDS-PAGE, blotted onto nitrocellulose membrane, and probed with the same antibodies as described above.

The binding affinity of CD11c scFv and rH9HA-CD11c scFv to the chicken APC receptors was characterised using western blot analysis of the chicken splenocyte extract. Briefly, chicken splenocytes were stimulated with 200 ng/mL of LPS (Merck Life Science, Darmstadt, Germany) for 24 h. The splenocytes were centrifuged, resuspended in 300 μL of NP-40 lysis buffer and vortexed. This was followed by incubation at room temperature for 30 min with constant vortexing. The splenocytes were then centrifuged at 4600 rpm for 10 min and the supernatant was harvested and analysed using western blot. Both the primary and secondary antibodies were diluted in phosphate buffer saline (PBS) containing 0.1% tween 20 (PBS-T) and 1% milk powder (Marvel). The primary antibodies used were 1 μg/mL of CD11c mAb or equimolar concentration of CD11c scFv and rH9HA-CD11c scFv. The secondary antibodies used were goat anti-mouse (1:10000, LICOR biosciences, Lincoln, NE, USA, primary antibody: CD11c mAb) or anti-V5 HRP (1:1000, Bio-Rad, primary antibody: CD11c scFv or rH9HA-CD11c scFv). For the latter, 3,3′-diaminobenzidine (DAB) substrate was added and development of bands on the membrane was observed.

### 2.6. Flow Cytometry

Briefly, 1 × 10^5^ chicken splenocytes were stimulated with 200 ng/mL LPS (Merck Life Science) for 24 h. The splenocytes were centrifuged and resuspended in 50 μL of FACS buffer (PBS with 1% Bovine Serum Albumin (BSA) containing 5 μg of CD11c scFv antibodies for 45 min at 4 °C. After the incubation with CD11c scFv antibodies, the splenocytes were washed with 150 μL of FACS buffer and resuspended in 100 μL of FACS buffer containing fluorescein isothiocyanate (FITC) conjugated anti-V5 tag secondary antibody (1:200, Bio-Rad) and incubated in dark for 30 min at 4 °C. Alternatively, if CD11c mAb (10 μg/mL) was used as a primary antibody, the cells were incubated for 20 min at 4 °C, followed by FITC conjugated goat anti-mouse secondary antibody (1:200, Bio-Rad) for 30 min at 4 °C. The labelled splenocytes were fixed with 50 μL of 1% paraformaldehyde (PFA) for 20 min in dark. The plates were read the next day using MACSQuant flow cytometer and analysed with FCS Express 6 software.

### 2.7. Bis[sulfosuccinimidyl] Suberate (BS3) Cross-Linking

The oligomeric structure of the recombinant H9HA containing trimerisation foldon domain was determined using BS3 (Life Technologies) cross-linking assay as described elsewhere [35] with the following modifications. Briefly, 15 µg recombinant protein was incubated at room temperature in the presence of BS3 (final concentration 10 mM) for one hour. Cross-linking was stopped by the addition of 1M Tris-HCl pH 8.0 to a final concentration of 50 mM. The cross-linked products were separated on SDS-PAGE gel under reducing conditions, blotted and immunodetected using anti-H9HA monoclonal antibody [36].

### 2.8. Preparation and Stimulation of Chicken Splenocytes

Splenocytes were prepared from the spleens of 3 weeks old SPF chickens (Rhode Island Red, Roslin) via density gradient centrifugation by using Histopaque 1083 (Merck Life Science) according to the manufacturer’s protocol. Briefly, spleens were mashed through 100 µm cell strainer (Merck Life Science) and suspended in complete Roswell Park Memorial Institute 1640 (RPMI) medium containing 10% FBS and 0.1% penicillin and streptomycin. This was followed by layering with Histopaque 1083 (Merck Life Science) and centrifuging at 400× *g* for 30 min. The splenocytes were then harvested from the ‘buffy coat’ interface in the density gradient and resuspended in a fresh complete RPMI media. About 2 × 10^6^ cells were plated on each well of 24 well plate and treated with 10 μg of rH9HA or 14 μg of rH9HA-Dec205/CD11c scFv (containing 10 μg rH9HA according to the molecular weight) or 10 μg of scFv or 10 μg of control scFv (anti-H9HA). All cells were stimulated for 5, 22, and 30 h in vitro at 41 °C.

### 2.9. RNA Extraction from Chicken Splenocytes and Quantitative Reverse Transcription PCR (qRT-PCR)

RNA was extracted from the stimulated splenocytes using RNeasy kit (Qiagen, Hilden, Germany) according to the manufacturer’s protocol. The qRT-PCR experiments were performed using Superscript III Platinum One-Step qRT-PCR kit (Life Technologies) as per the manufacturer’s protocol in a 7500 FAST ABI RT-PCR thermocycler (Applied Biosystems, Waltham, MA, USA). Sequences of primers and probes used for qRT-PCR are shown in Table S1. Cycling conditions used were as follows: (i) 5 min hold step at 50 °C, (ii) 2 min hold step at 95 °C, (iii) 40 cycles of 95 °C (3 s) and 60 °C (30 s). Cycle threshold (CT) values were obtained using 7500 software v2.3 and exported to Microsoft excel for further analysis. Mean CT values were calculated from triplicate data. Negative controls were included within each plate to determine any unspecific amplification or contamination. Data were calculated using 2-∆∆CT approach (n-fold change compared to the media only control group) and reported as values normalised to the expression level of a housekeeping gene RPLPO1 (Ribosomal phosphoprotein lateral stalk subunit PO). Out of the three reference genes (RPLPO-1, RPL13 and 28S) selected for normalisation, RPLPO1 was the most stable gene across the samples hence, chosen for normalisation.

### 2.10. Haemagglutination Assay and Haemagglutination Inhibition (HI) Assay

Haemagglutination assay was performed as previously described [37]. For HI assay, two-fold serial dilution of the serum was prepared by mixing 25 μL of serum with 25 μL PBS. The diluted serum was then incubated with 4 HA units of UDL 01/08 H9N2 virus for 1 h at 37 °C. This was followed by addition of 50 μL of 1% chicken red blood cells (RBCs) and further incubation for 1 h at 37 °C. HI titres were expressed as reciprocal of the highest dilution of serum that causes total inhibition of 4 HA units of virus haemagglutination activity.

### 2.11. Chicken Vaccination and Blood Sample Collection

Groups of 7-day-old SPF chickens (Dekalb White, Henry Stewart & Co. Ltd, Fakenham, UK) (n = 8 per group) were immunised with 2.8, 28, and 49 µg of rH9HA-Dec205 scFv or rH9HA-CD11c scFv equivalent to 2, 20, and 35 µg of rH9HA (equimolar concentration). Additionally, we included one group of chickens (n = 8 per group) immunised with inactivated H9N2 vaccine (UDL 01/08, ~1 × 10^8^ EID_50_ (50% egg infectious dose) per mL). All vaccines were formulated in MontanideTM ISA 71R VG (Seppic, Courbevoie, France) as water-in-oil emulsion according to the manufacturer’s protocol. Vaccines were administered to the chickens via subcutaneous route, at the back of the neck. Control groups were immunised withPBS. All vaccinated groups received a booster dose at 14-day-old. In all cases, blood samples were collected from the wing vein 6, 14, 21, and 28 days post primary vaccination (ppv).

### 2.12. Measurement of Serum IgM, IgY and IgA Anti-HA Antibody Levels

The anti-HA specific IgM, IgY, and IgA antibodies in the serum were determined by ELISA assay. Briefly, 96 well maxisorp ELISA plates (Life Technologies) were coated with 1 μg of rH9HA protein diluted in carbonate buffer (15 mM NaCO_3_, 35 mM NaHCO_3_, 3 mM NaN_3_) and incubated overnight at 4 °C. Protein coated plates were blocked at room temperature with 5% milk powder (Marvel) in PBS-T for 1 hour. Plates were washed thrice with PBS-T. Chicken sera were diluted (1:200) in PBS-T buffer containing 1% milk powder (Marvel). The plates were then incubated with the diluted sera (50 μL/well) at room temperature for 1 h. This was followed by further incubation for 1 h at room temperature with 50 μL per well of goat anti-chicken IgM, IgY and IgA antibodies conjugated to HRP (1:3000, Abcam, Cambridge, UK). Plates were washed 4× with PBS-T then 100 μL per well of TMB substrate (Life Technologies) was added for 10 min. The reaction was stopped using 2M H_2_SO_4_ (100 μL/well) and read at wavelength 450 nm in ELx808 absorbance microplate reader (BioTek, Winooski, VT, USA). A standard reference serum (serum collected from 35-day-old chicken challenged with UDL 01/08 H9N2 virus was included in all assays. The amount of anti-HA IgM, IgY or IgA antibodies were expressed as sample to reference ratio (relation of absorbance of tested serum sample to absorbance of the reference serum).

### 2.13. Plaque Assay

Plaque assay was performed to titrate the virus from egg allantoic fluid. Briefly, confluent MDCK cells were washed with PBS then infected with serial dilutions of influenza virus. Virus inoculum was removed from the cells after 1 h. Cells were then overlaid by plaque assay overlay media (1x DMEM, 0.21% BSA, 1 mM L-glutamate, 0.15% sodium bicarbonate, 10 mM Hepes, 0.1% penicillin G/streptomycin, 0.6% *w*/*v* purified agar (Oxoid) containing 2 μg/mL of N-tosyl-L-phenylalanyl chloromethyl ketone (TPCK) trypsin (Life Technologies). After solidification of the overlay media, plates were inverted and kept in incubator at 37 °C for 3–4 days. When plaques were visible agar plugs were removed and cells were stained with 1 mL of 0.1% crystal violet solution (Merck Life Science).

### 2.14. Virus Micro-Neutralisation (MNT) Assay

MDCK cells were pre-seeded into the 96 well plates to reach 90–95% confluency. The chicken serum was inactivated at 56 °C for 30 min, and an initial dilution of 1:200 was made in PBS buffer. This was followed by two-fold serial dilution in triplicates and mixing of 90 μL of diluted serum with 90 μL of UDL 01/2008 H9N2 virus (total 180 μL/well) containing 150 TCID_50_. The serum-virus mixture was incubated at 37 °C for 1 h. Cells were washed with PBS and inoculated with sera-virus mixture for 1 h at 37 °C. After the incubation, cells were washed with PBS and serum free DMEM media containing 2 µg/mL TPCK trypsin was added (100 μL/well). Cells were left at 37 °C for 3–4 days. After 3–4 days medium was removed, and cells were stained in 50 μL of 0.1% crystal violet solution (Merck Life Science) for 30 min. Virus MNT titres were expressed as reciprocal of the highest dilution of antiserum that blocks the virus infectivity in cultured cells inoculated with 150 TCID_50_.

### 2.15. Statistical Analysis

Results are expressed as the mean ± standard deviation (SD). Statistical significance (*p*-values) was determined followed by post hoc Tukey’s multiple comparison test using Prism 8.3.0 (GraphPad Software, San Diego, CA, USA). Differences were considered statistically significant if *p* < 0.05.

## 3. Results

### 3.1. Expression and Purification of the Recombinant Proteins

**Figure 1:**
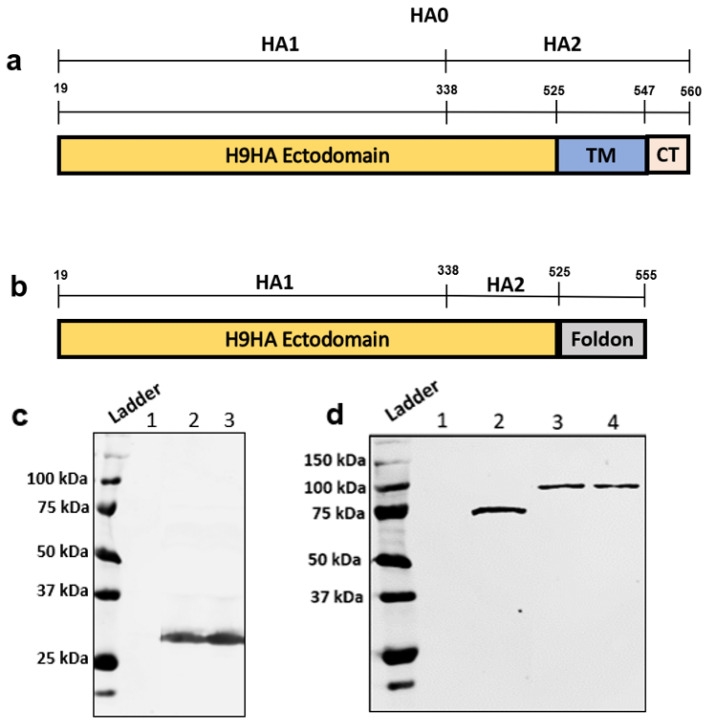
Schematic representation of the H9HA construct and His tag purification of the recombinant proteins (**a**) Full length H9HA precursor (HA0); HA0: 1–560 amino acids (aa), HA1: 19–338 aa, HA2: 339–560 aa, TM=Transmembrane domain (525–547 aa) CT=Cytosolic Tail domain (548–560 aa) (**b**) Soluble H9HA construct. The soluble H9HA construct was generated by removing the TM and CT domains (525–560 aa) and fusing the C-terminus of HA to 30 aa long trimerisation foldon domain of the protein fibritin from T4 bacteriophage (**c**) His tag purification of scFv antibody. The size of the purified protein is 30 kDa. Lane 1: Control supernatant from the untransfected cells Lane 2: Dec205 scFv Lane 3: CD11c scFv (**d**) His tag purification of rH9HA and rH9HA-scFv. The size of the purified proteins is about 70 kDa and 100 kDa respectively. Lane 1: Control supernatant from the untransfected cells Lane 2: rH9HA Lane 3: rH9HA-Dec205 scFv Lane 4: rH9HA-CD11c scFv. For the purification of the recombinant proteins, the harvested S2 cell culture supernatants containing recombinant proteins bound to the metal ions (copper sulphate was used as an inducer of metallothionein promoter) were loaded onto Profinity™ IMAC column (Bio Rad). Proteins were eluted with elution buffer containing 50 mM NaH_2_PO_4_, 300 mM NaCl and 50 mM imidazole. The purified proteins were analysed by 10% SDS-PAGE followed by Coomassie staining.

The soluble H9HA protein was generated by removing the transmembrane domain and replacing it with 30 amino acid trimerisation foldon domain of fibritin protein from bacteriophage T4 [32] at C-terminus (Figure 1a,b). Furthermore, rH9HA was recombinantly fused to scFv antibodies targeting chicken Dec205 and CD11c. The Dec205/CD83 scFv, rH9HA, and rH9HA-Dec205/CD83 scFv proteins were successfully expressed in *Drosophila melanogaster* S2 cells and secreted as indicated by the SDS-PAGE of the purified proteins from the culture supernatant (Figure 1c,d). Subsequent purification of the recombinant protein containing supernatants by His-tag affinity chromatography yielded proteins with molecular weights of approximately 30 kDa for scFv, 70 kDa for rH9HA, and 100 kDa for rH9HA-Dec205/CD11c scFv. Moreover, the purified rH9HA protein migrated as a single polypeptide of about 70 kDa on SDS-PAGE under reducing condition. This indicates that the recombinant rH9HA protein was expressed as HA precursor (HA0). This observation is in line with previous reports of soluble HA expression as precursor HA0 [35,38]. Based on the recovered purified proteins, the expression levels of the recombinant proteins were estimated to be between 10–20 mg per litre of the culture supernatant.

### 3.2. rH9HA Can Trimerise and Retain the Haemagglutination Activity

**Figure 2:**
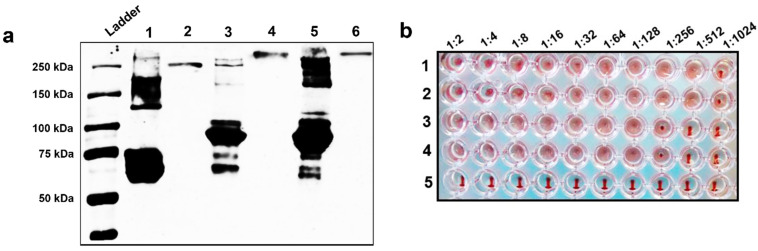
BS3 crosslinking experiment and haemagglutination assay to determine the oligomeric form and activity of recombinant H9HA ectodomain with foldon respectively (**a**) BS3 crosslinking experiment. Lane 1: rH9HA without BS3; Lane 2: rH9HA with 10 mM BS3; Lane 3: rH9HA-Dec205 scFv without BS3; Lane 4: rH9HA-Dec205 scFv with 10 mM BS3; Lane 5: rH9HA-CD11c scFv without BS3; Lane 6: rH9HA-CD11c scFv with 10 mM BS3. About 15 µg of the recombinant protein was mixed with BS3 to a 10 mM final concentration and incubated for 1 h at room temperature. The cross-linking reaction was stopped by the addition of 1 M Tris-HCl pH 8.0 to a final concentration of 50 mM and incubated for 15 min at room temperature. After cross-linking, proteins were separated on 8% SDS-PAGE under reducing conditions, blotted and immunodetected using anti-H9HA monoclonal antibody. (**b**) Haemagglutination assay to test the activity of recombinant H9HA with foldon. 1. UDL 01/08 H9N2 virus; 2. rH9HA; 3. rH9HA-Dec205 scFv; 4. rH9HA-CD11c scFv; 5. Negative control (PBS). For haemagglutination assay, a two-fold serial dilution of 35 μg of the recombinant HA proteins was carried out in 96-well plates. 50 µL of 1% chicken RBCs was added. The plates were incubated at 4 °C for one hour and the highest dilution of the protein causing the agglutination of the RBCs was noted.

The HA protein is trimeric in nature and this trimeric structure is needed to retain the biological activity of the protein [38]. The oligomerisation state of H9HA ectodomain containing trimerisation foldon domain was determined by cross-linking using a BS3 cross-linker. BS3 is a water-soluble cross-linker which reacts with primary amines of target proteins to form stable amide bond [39]. When multimeric proteins are exposed to BS3, each subunit will be cross-linked together with the formation of amide bonds. This provides direct evidence for their close proximity [39]. This also helps to stabilise the structure of oligomers [40], allowing them to be analysed on SDS denaturing gels for Western blot analysis. The recombinant rH9HA and rH9HA-Dec205/CD11c scFv proteins were incubated with BS3 cross-linker and the cross-linked products were separated on SDS-PAGE gel under reducing and denaturing conditions, blotted and immunodetected using anti-H9HA monoclonal antibody (Figure 2a). Without BS3 crosslinking, three species; monomer, dimer, and trimer were observed (major band at monomer: lane 1, lane 3, and lane 5 corresponding to about 70 kDa and 100 kDa for rH9HA and rH9HA-Dec205/CD11c scFv respectively). When cross-linked, stable trimeric form was observed (lane 2, lane 4, and lane 6 corresponding to about 210 kDa and 300 kDa for rH9HA and rH9HA-Dec205/CD11c scFv respectively). This indicates that the native structure of recombinant H9HA protein with foldon is a trimer, and three molecules of scFv antibodies are attached to one trimeric H9HA protein. 

Next, the biological activity of rH9HA and rH9HA-Dec205/CD83 scFv proteins was determined using haemagglutination assay (Figure 2b). The rH9HA protein, both on its own and when fused to Dec205/CD11c scFv antibodies was able to agglutinate chicken RBCs retaining its haemagglutination activity. This demonstrates that the modifications of H9HA protein does not affect its native structure and ability to bind sialic acid receptors on chicken RBCs.

### 3.3. scFv Antibodies Can Retain Their Function after Fusion to rH9HA 

**Figure 3:**
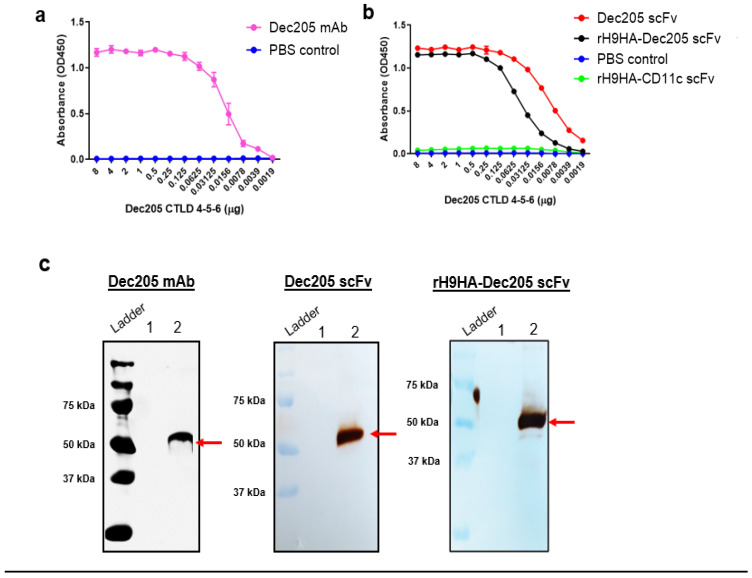
Characterisation of Dec205 mAb, Dec205 scFv and rH9HA-Dec205 scFv using ELISA and western blot assays (**a**,**b**) ELISA analysis for testing the ability of Dec205 mAb, Dec205 scFv and rH9HA-Dec205 scFv to bind to chicken Dec205 CTLD 4-5-6. About 8 μg of purified chicken Dec205 CTLD 4-5-6 was coated onto each well of the ELISA plate, a two-fold serial dilution was carried out and the plate was incubated overnight for 4 °C. For detection, the plates were incubated 1 μg/mL of Dec205 mAb or equimolar concentration of Dec205 scFv and rH9HA-Dec205 scFv. This was followed by incubation with goat anti-mouse HRP secondary antibody for Dec205 mAb (1:1000) and HRP-conjugated anti-V5 secondary antibody for Dec205 scFv and rH9HA-Dec205 scFv (1:1000). The colorimetric detection was carried out by adding TMB substrate and absorbance at 450 nm was recorded. No significant differences were observed in the binding between Dec205 scFv and rH9HA-Dec205 scFv. (**c**) Western blot analysis for testing the ability of Dec205 mAb, Dec205 scFv and rH9HA-Dec205 scFv to detect chicken Dec205 CTLD 4-5-6. In all cases, lane 1 represents untransfected cell supernatant control whereas lane 2 represents chicken Dec205 CTLD 4-5-6 (red arrow). The chicken Dec205 CTLD 4-5-6 was run on 10% SDS-PAGE, blotted onto nitrocellulose membrane, and immunodetected using the same primary and secondary antibodies as that of the ELISA assay.

The rH9HA is about 70 kDa in size hence, the fusion of such a large molecule to scFv antibodies could jeopardise the function of the antibodies. Here, ELISA and Western blot assays were used to assess the function of scFv antibodies fused to rH9HA. Plates were coated with purified chicken Dec205 CTLD 4-5-6. Dec205 mAb was included in the ELISA assay as a positive control (Figure 3a). Furthermore, rH9HA-CD11c scFv was used as a negative control to assess any unspecific binding to chicken Dec205 CTLD 4-5-6. No unspecific binding by rH9HA-CD11c scFv was observed indicating that any positive signal is purely due to Dec205 scFv binding. Moreover, both Dec205 scFv and rH9HA-Dec205 scFv were able to detect and bind to Dec205 CTLD 4-5-6 (Figure 3b). There were no significant differences in the binding between Dec205 scFv and rH9HA-Dec205 scFv. However, the binding to Dec205 CTLD 4-5-6 by rH9HA-Dec205 scFv was lower than Dec205 scFv at lower concentration (<0.125 μg) of Dec205 CTLD 4-5-6. In addition, Western blot analysis was also carried out to verify the results obtained from ELISA assay. Dec205 mAb, Dec205 scFv, and rH9HA-Dec205 scFv showed a single band at approximately 53 kDa, which is the expected molecular weight of chicken Dec205 CTLD 4-5-6 (Figure 3c).

**Figure 4:**
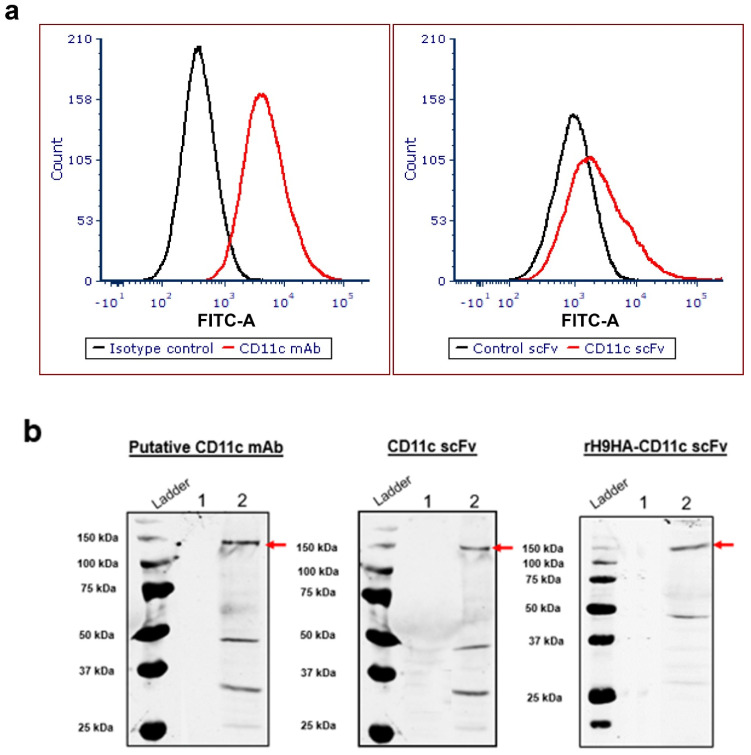
Characterisation of CD11c mAb, CD11c scFv, and rH9HA-CD11c scFv. (**a**) Staining of chicken splenocytes by putative CD11c mAb and CD11c scFv using flow cytometry. About 1 × 105 chicken splenocytes were stimulated with 200 ng/mL of LPS for 24 h. The splenocytes were centrifuged and incubated with 1 μg/mL putative CD11c mAb for 20 min at 4 °C or 3 μg/mL of CD11c scFv for 45 min at 4 °C. This was followed by incubation with secondary antibodies (FITC conjugated goat anti-mouse secondary antibody (1:200) if CD11c mAb was used or FITC conjugated anti V5 tag (1:100 dilution) if CD11c scFv was used) and incubated in dark for 30 min at 4 °C. The labelled splenocytes were fixed with 50 μL of 1% PFA for 20 min in dark. The plates were read the next day using MACSQuant flow cytometer and analysed with FCS Express 6 software. (**b**) Detection of CD11c receptor protein from the chicken splenocyte extract by putative CD11c mAb, CD11c scFv, and rH9HA-CD11c scFv. In all cases, lane 1 represents *Drosophila melanogaster* S2 cell extract (control) and lane 2 represents chicken splenocyte extract (red arrow). The chicken splenocyte extract was run on 8–10% SDS-PAGE, blotted onto nitrocellulose membrane and immunodetected using either 1 μg/mL of CD11c mAb or equimolar concentration of CD11c scFv and rH9HA-CD11c scFv.

The immunogen used for the generation of hybridoma for chicken CD11c (clone: IAH 8F2) could not be identified (unpublished data). However, it has been reported that thechicken CD11c mAb (clone: IAH 8F2) stains chicken DCs and have been used by several groups to enrich and phenotype these cells [41]. Furthermore, CD11c mAb (clone: IAH 8F2) has been applied to stain chicken bursal secretory dendritic cells [27], distinguish isolated splenic DC-like cells from KUL01+ macrophages [42], and also for the phenotypic analysis of chicken bone marrow derived DCs [28]. Chicken splenocytes were also stained by putative CD11c mAb and CD11c scFv (Figure 4a). Probing of chicken splenocytes extract with putative CD11c mAb, CD11c scFv, and rH9HA-CD11c scFv in western blot analysis yielded three bands with approximate molecular weights of 150, 50, and 35 kDa (Figure 4b). The band corresponding to 150 kDa represents expected molecular weight of CD11c receptor protein [43]. The identity of the bands corresponding to 50 kDa and 35 kDa is unknown. It has been reported that the putative CD11c mAb (clone: IAH 8F2) reacts with an uncharacterised cell surface heterodimer protein on chicken monocytes/macrophages or natural killer cells [41].

### 3.4. rH9HA-CD11c scFv Is Better at Stimulating Chicken Splenocytes to Produce Different Cytokines

**Figure 5:**
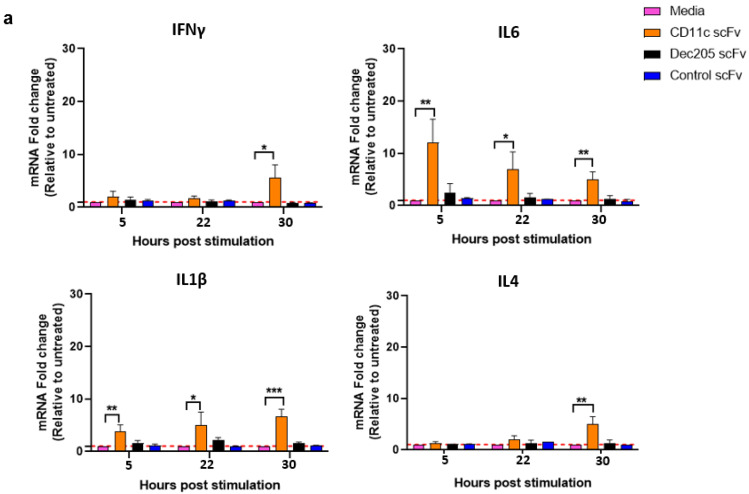

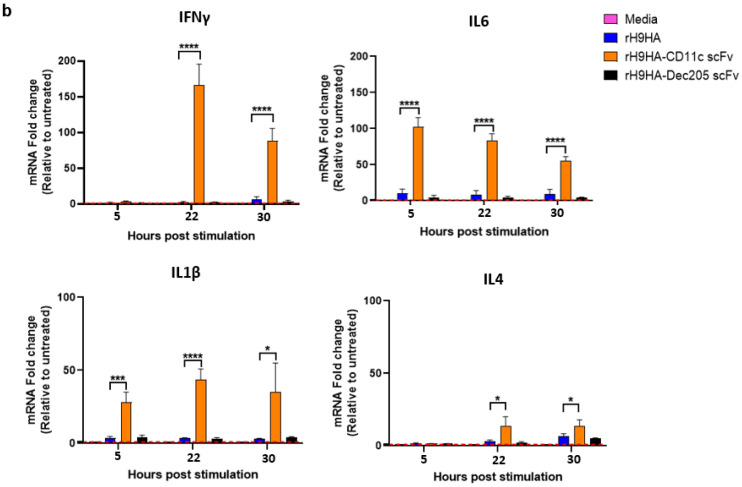
qRT-PCR analysis of IFNγ, IL6, IL1β and IL4 production by splenocytes upon stimulation with scFv, rH9HA, and rH9HA-scFv. (**a**) Analysis of cytokine mRNA levels in splenocytes stimulated with Dec205 scFv and CD11c scFv. (**b**) Analysis of cytokine mRNA levels in splenocytes stimulated with rH9HA, rH9HA-Dec205 scFv, and rH9HA-CD11c scFv. Splenocytes were isolated from the spleen of 3 weeks old SPF chickens using Histopaque 1083 and stimulated with 10 μg of scFv/rH9HA/rH9HA-scFv for 5, 22, and 30 h in vitro. Stimulated splenocytes were harvested for RNA extraction and the expression levels of the respective cytokines were measured by qRT-PCR. Data were calculated using 2-∆∆CT approach (n-fold change compared to the media only control group) and reported as values normalised to the expression level of a housekeeping gene RPLPO1. Data are presented as mean + SD and analysed by one-way ANOVA followed by Tukey’s multiple comparison test. **** *p* < 0.0001, *** *p* < 0.001, ** *p* < 0.01, * *p* < 0.05. The data represent three independent experiments.

To determine the immunogenic potential of rH9HA-Dec205 scFv and rH9HA-CD11c scFv, splenocytes were isolated from SPF chickens and treated with the respective proteins for 5 h, 22 h and 30 h in vitro. The production of different cytokines like IFNγ, IL6, IL4, and IL1β was assessed using qRT-PCR. With regards to scFv, Dec205 scFv was unable to induce the production of all the tested cytokines. However, CD11c scFv stimulation showed significantly higher mRNA levels of IFNγ (*p* < 0.05 for 30 h post stimulation (ps)), IL6 (*p* < 0.01 for 5 h and 30 h ps, *p* < 0.05 for 22 h ps), IL1β (*p* < 0.01 for 5 h ps, *p* < 0.05 for 22 h ps, *p* < 0.001 for 30 h ps), and IL4 (*p* < 0.01 for 30 h ps) compared to control scFv (Figure 5a).

Similarly, rH9HA-Dec205 scFv was also unable to induce the production of tested cytokines. Interestingly, stimulation with rH9HA-CD11c scFv showed an overall higher mRNA levels of all the cytokines tested compared to CD11c scFv. A significantly higher mRNA levels of IFNγ (*p* < 0.0001 for 22 h and 30 h ps), IL6 (*p* < 0.0001 for 5 h, 22 h and 30 h ps), IL1β (*p* < 0.001 for 5 h ps, *p* < 0.0001 for 22 h ps, *p* < 0.05 for 30 h ps), and IL4 (*p* < 0.05 for 22 h and 30 h ps) was observed with rH9HA-CD11c stimulation compared to rH9HA (Figure 5b). Furthermore, the induction of IL6 and IL1β cytokines occurred earlier (after 5 h ps) whereas IFNγ and IL4 were induced later (after 22 h ps). These results suggest that rH9HA-CD11c scFv is better than rH9HA-Dec205 scFv in inducing cytokine expression in chicken splenocytes in vitro.

### 3.5. Immunisation with rH9HA-Dec205 scFv and rH9HA-CD11c scFv Induces Higher Antibody Response in Chickens

**Figure 6:**
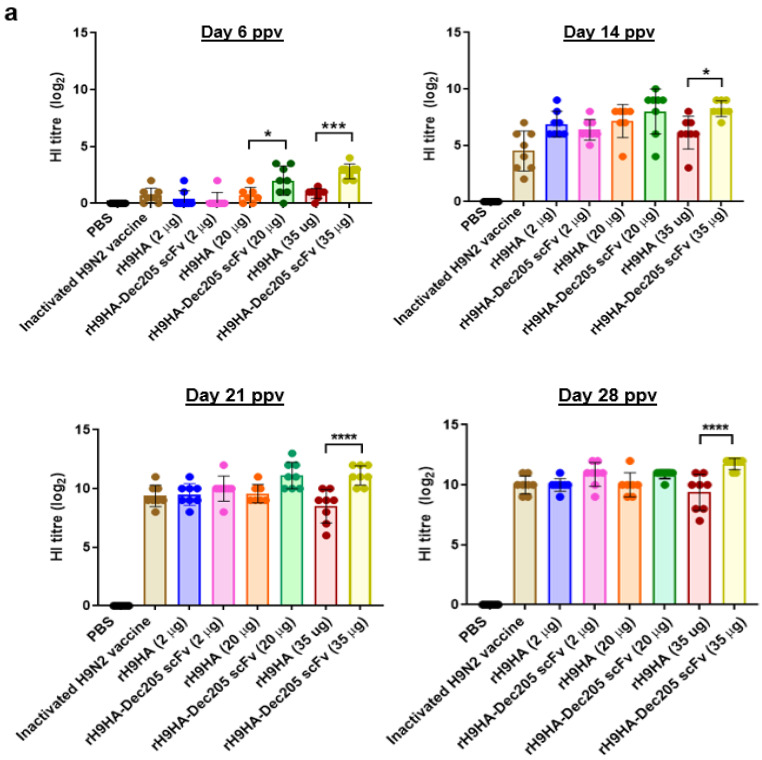

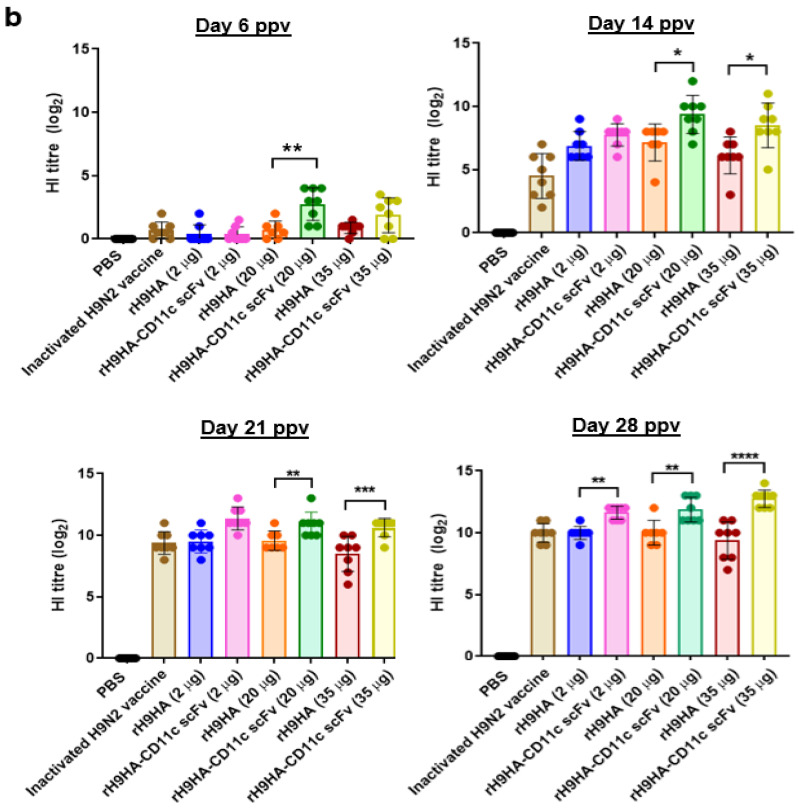
Analysis of HI antibodies in the serum of vaccinated chickens. (**a**) HI antibody titres in chickens vaccinated with rH9HA, rH9HA-Dec205 scFv, and inactivated H9N2 vaccine. (**b**) HI antibody titres in chickens vaccinated with rH9HA, rH9HA-CD11c scFv, and inactivated H9N2 vaccine. Groups of 7-day-old chickens (n = 8 per group) were immunised with 2.8, 28, and 49 µg of rH9HA-Dec205/CD11c scFv equivalent to 2, 20, and 35 µg of rH9HA (equimolar concentration). Boost vaccination was given on day 7 post primary vaccination. The chickens were bled on day 6, 14, 21, and 28 post primary vaccination (ppv). The highest dilution of serum inhibiting the agglutination of chicken RBCs by H9N2 virus (UDL 01/08) was recorded. Data are presented as mean ± SD and analysed by one-way ANOVA followed by Tukey’s multiple comparison test. **** *p* < 0.0001, *** *p* < 0.001, ** *p* < 0.01, * *p* < 0.05.

A standard HI assay was used to test the immunogenicity of rH9HA-Dec205 scFv and rH9HA-CD11c scFv (Figure 6). The serum antibody titres were measured at 6, 14, 21, and 28 days ppv. The chickens vaccinated with rH9HA-Dec205 scFv were able to induce significantly higher HI antibody titres compared to rH9HA group as early as day 6 ppv with the 20 μg (*p* < 0.05) and 35 μg (*p* < 0.001) vaccine doses. However, after day 14 ppv significant differences in HI antibody titres between rH9HA and rH9HA-Dec205 scFv groups were observed only with the 35 μg dose (day 14 ppv: *p* < 0.05, day 21 ppv: *p* < 0.0001, day 28 ppv: *p* < 0.0001) (Figure 6a). With regards to chickens vaccinated with rH9HA-CD11c scFv, significantly higher HI antibody titres were observed with the 20 μg dose on day 6 ppv compared to rH9HA group (*p* < 0.01). Furthermore, rH9HA-CD11c scFv also produced significantly higher HI antibody titres compared to rH9HA with the 20 μg (day 14 ppv: *p* < 0.05, day 21 ppv: *p* < 0.01, day 28 ppv: *p* < 0.01) and 35 μg ppv (day 14 ppv: *p* < 0.05, day 21 ppv: *p* < 0.001, day 28 ppv: *p* < 0.0001) doses from day 14 ppv (Figure 6b). Moreover, no significant differences were observed between the 2, 20, and 35 μg doses of vaccination in rH9HA immunised groups on all the time points tested. However, with rH9HA-Dec205 scFv and rH9HA-CD11c scFv groups higher HI antibody titres were produced with the 20 μg and 35 μg doses compared to the 2 μg dose on most of the time points tested. In addition, the 20 μg and 35 μg doses of rH9HA-Dec205 scFv and rH9HA-CD11c scFv groups showed significantly higher HI antibody titre compared to the inactivated H9N2 vaccine group (day 6 ppv: *p* < 0.05, day 14 ppv: *p* < 0.001, day 21 ppv: *p* < 0.05, day 28 ppv: *p* < 0.01).

**Figure 7:**
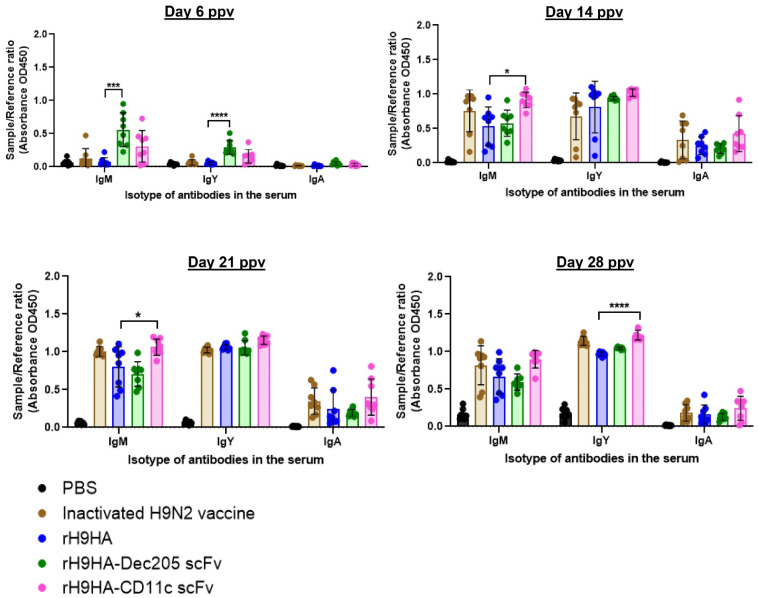
HA-specific IgM, IgY, and IgA antibodies in the serum of chickens immunised with 35 μg dose of rH9HA, rH9HA-Dec205 scFv, rH9HA-CD11c scFv, and inactivated H9N2 vaccines. The HA-specific isotypes of the antibodies were determined in 200-fold diluted sera collected on day 6, 14, 21, and 28 post primary vaccination (ppv) by ELISA. The plates were coated with 1 μg of recombinant H9HA protein overnight at 4 °C. For detection, the plates were incubated with respective sera for one hour at room temperature. This was followed by further incubation for one hour with 1:3000 diluted anti-chicken IgM, IgY, and IgA antibodies. The colorimetric detection was carried out by adding TMB substrate and absorbance at 450 nm was recorded. The amount of HA specific IgM, IgY and IgA antibodies were expressed as sample to reference ratio (relation of absorbance of tested serum sample to absorbance of the reference serum). Data are presented as mean ± SD and analysed by one-way ANOVA followed by Tukey’s multiple comparison test. **** *p* < 0.0001, *** *p* < 0.001, * *p* < 0.05.

Furthermore, ELISA assay was also performed to determine the total anti-HA IgM, IgY, and IgA antibodies in the serum of chickens immunised with 35 μg dose of vaccines at 6, 14, 21, and 28 days ppv (Figure 7). Both rH9HA-Dec205 scFv and rH9HA-CD11c groups showed higher amount of anti-HA IgM and IgY antibodies compared to anti-HA IgA antibodies in the immunised serum. The chickens in rH9HA-Dec205 scFv group showed significantly higher anti-HA IgM (*p* < 0.001) and IgY (*p* < 0.0001) antibodies compared to rH9HA on day 6 ppv. However, after day 6 ppv no differences were observed in the anti-HA antibodies between rH9HA and rH9HA-Dec205 scFv groups. On the contrary, rH9HA-CD11c showed no differences in the anti-HA antibodies compared to rH9HA on day 6 ppv. However, significantly higher anti-HA IgM (*p* < 0.05) and IgY (*p* < 0.0001) antibodies were observed in rH9HA-CD11c scFv group compared to rH9HA group on day 14 ppv/day 21 ppv and day 28 ppv, respectively.

**Figure 8:**
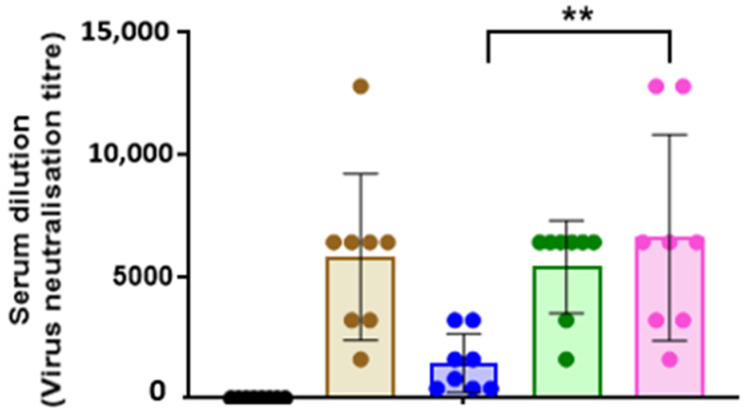
Virus neutralising antibody titre in the serum of chickens vaccinated with 35 μg dose of rH9HA, rH9HA-Dec205 scFv, rH9HA-CD11c scFv, and inactivated H9N2 vaccines. Virus micro-neutralisation assay was carried out on day 28 post primary vaccination serum samples. The serum was first inactivated at 56 °C for 30 min and two-fold serum dilutions were made. Then, the inactivated serum was incubated with 150 TCID_50_ of H9N2 virus (UDL 01/08) for 1 h at 37 °C. The virus/serum mixture was then added onto the MDCK cells and further incubated for 1 h at 37 °C. This was followed by the removal of the virus-serum mixture from the cells and incubation with fresh media containing 2 μg/mL TPCK trypsin for about 3 days. The virus neutralisation titre was expressed as the reciprocal of the highest serum dilution at which virus infection is blocked and the cells survive. Data are presented as mean ± SD and analysed by one-way ANOVA followed by Tukey’s multiple comparison test. ** *p* < 0.01.

In addition, the virus MNT assay was also conducted with the immunised serum from the 35 μg vaccine dose at day 28 ppv (Figure 8). The virus MNT assay is believed to be more sensitive than HI assay, and it measures serum antibodies that can block the cytopathic effects of the virus [44]. There were no significant differences in the virus MNT antibody titres between rH9HA and rH9HA-Dec205 scFv groups. However, rH9HA-CD11c scFv group produced significantly higher virus MNT antibody titre compared to rH9HA group (*p* < 0.01).

## 4. Discussion

Vaccines have been established as additional measures to control poultry diseases. Moreover, effective vaccines have been responsible for a remarkable reduction in disease loss during the past decades in commercial flocks [45]. The majority of commercial poultry vaccines lack the ability to induce optimal immunity hence, there is a need to improve their efficacy. One strategy of enhancing the efficacy of vaccines is to selectively deliver antigens to professional APCs, such as dendritic cells, macrophages, and B cells [9]. In this study, we developed vaccines based on targeted delivery of HA antigen of H9N2 AIV. The H9HA protein was modified for soluble expression in *Drosophila melanogaster* S2 cells by eliminating the TM domain and fusing the C-terminus of H9HA protein to trimerisation foldon domain of T4 bacteriophage fibritin protein [32]. This particular trimerisation foldon domain has been used previously for high level of secretory expression of stabilised native trimeric H9HA protein [37]. The modified H9HA protein was then recombinantly fused with scFv portion of the antibody that have binding specificity for the chicken Dec205 and CD11c. Dec205 expression is primarily restricted to DCs whereas CD11c are expressed on DCs, macrophages, NK cells, and activated T cells [8]. The modified H9HA protein with foldon was able to trimerise into its native structure, retaining its function to agglutinate chicken RBCs. Furthermore, the fusion of rH9HA with scFv antibody does not seem to affect the functional activity of both HA as well as the scFv antibodies. The recombinant rH9HA-Dec205/CD11c scFv was able to agglutinate chicken RBCs in HA assay, and the scFv antibodies fused to H9HA were able to detect and bind to their respective receptor proteins in ELISA and Western blot assays. 

Vaccine formulations often include PAMPs like LPS, CpG oligonucleotides or flagellin to improve and accelerate both humoral and cell-mediated immune responses [46]. Such PAMPs stimulate the production of pro-inflammatory cytokines/chemokines that can increase host’s ability to eliminate the pathogen. However, there has always been concerns over the safety of PAMPs in vaccines, particularly the possibility that they might increase susceptibility to toxic shock and autoimmune disease [47]. Thus, the quest of finding a better alternative to PAMPs to improve the vaccine efficacy is long on-going. Here, we evaluated the priming effects of Dec205 scFv, CD11c scFv, rH9HA-Dec205 scFv, and rH9HA-CD11c scFv on chicken splenocytes in vitro, by assessing the mRNA expression levels of different cytokines. It was found that Dec205 scFv, rH9HA-Dec205 scFv, and rH9HA were unable to induce the production of any of the cytokines tested. On the other hand, CD11c scFv induced moderate upregulation of mRNA encoding pro-inflammatory cytokines like IFNγ, IL6, and IL1β. There was a higher upregulation of mRNA encoding cytokines IFNγ, IL6, IL1β, and IL4 by CD11c fused rH9HA. The differences observed in the ability of CD11c scFv and Dec205 scFv to stimulate chicken splenocytes for cytokine production could be attributed to the differential expression of the targeted receptors by different immune cells—e.g., CD11c is expressed not only on DCs but also on activated lymphocytes and macrophages—whereas Dec205 receptor is exclusively expressed on dendritic cells [17,27,48]. Previous study in mice has shown that CD11 Fab fused OVA is better at generating endogenous IFNγ producing CD4^+^ and CD8^+^ T cells in vivo compared to Dec205 Fab fused OVA [29]. Moreover, rH9HA-CD11c scFv was better at inducing higher mRNA levels of cytokines compared to CD11c scFv alone. It has been suggested that binding of antigen to antibody can result in the conformational changes in the antibody. This conformational change can enhance the uptake of antigen-antibody complexes by APCs compared to the free antibody hence, increasing the immunostimulatory potential of the antigen [49]. In addition, it is also possible that the observed stimulation effects could be attributed to the monomeric and trimeric forms of scFv in CD11c scFv and rH9HA-CD11c scFv constructs, respectively.

The results from the vaccination study suggested that serum HI antibody titres were higher for chickens vaccinated with 20 μg and 35 μg of rH9HA-Dec205 scFv and rH9HA-CD11c scFv compared to 2 μg of the respective vaccines. Furthermore, HI antibodies were produced as early as day 6 ppv with 20 μg and 35 μg of rH9HA-Dec205 scFv and rH9HA-CD11c scFv (only 20 μg) vaccine doses. An earlier study in chickens evaluated the targeting of H5HA antigen to Dec205 receptor using Dec205 mAb [16]. The study reported an increased serum antibody titre with 50 μg and 100 μg of the targeted proteins on day 14 ppv. A direct comparison between the previous and current study cannot be made since the previous study used Dec205 mAb instead of scFv, LPS as adjuvant and a single vaccination regime. It has been reported that inactivated virus vaccines are better at eliciting higher HI antibody titres compared to subunit vaccines [50,51]. Here, we observed that 20 µg and 35 µg doses of the recombinant rH9HA-Dec205 scFv and rH9HA-CD11c scFv produced higher serum HI antibodies compared to the inactivated virus vaccine. Interestingly, higher antibody responses were evoked by the recombinant vaccines without the need of DC stimulating adjuvants like LPS or CpG oligonucleotides, suggesting that the side effects of these adjuvants could be avoided by using TADV. Overall, the results from HI assay, anti-HA IgM, and IgY ELISA and virus MNT assays indicate that CD11c targeted rH9HA is better than Dec205 scFv targeted rH9HA in inducing higher antibody responses. Similar observation was made before where different targeting antibodies including those specific for mouse MHC class II, CD11a, CD11b, CD11c, Dec205, and CD40 were compared in vivo. CD11c targeting was found to be the most potent among all the receptors targeted [21]. Furthermore, another study demonstrated that OVA antigen targeted to CD11c was 3–4-fold more efficient in priming T cells for proliferation compared to Dec205 targeting in mice [22]. This remarkable adjuvant effect of anti-CD11c targeting was reported to be due to the enhanced germinal centre (GC) and extrafollicular (EF) plasma cell responses, which have direct stimulatory effect on the activation of antigen-specific B cells [21]. Moreover, the putative chicken CD11c mAb used to generate the targeting CD11c scFv in this study also binds to some uncharacterised proteins other than CD11c receptor protein on chicken splenocytes. Therefore, this could have influenced the cytokine expression levels and the antibody responses obtained with rH9HA-CD11c scFv. In future, it is important to characterise all the additional proteins bound by CD11c mAb (clone: IAH 8F2) as these could potentially have unknown effects in vivo. Some mammalian antigen targeting studies have shown to induce both humoral and cell mediated immunity (CMI) [29,52]. In this study, we also evaluated the CMI induced by rH9HA, rH9HA-Dec205/CD11c scFv by using IFNγ ELISPOT. However, due to the lack of optimal recall antigen, no CMI was observed with IFNγ ELISPOT assay (data not shown). Hence, further studies are required to investigate CMI induced by the respective vaccines. Nevertheless, we show that targeting H9HA antigen to chicken Dec205 and CD11c receptors enhances the vaccine immunogenicity, with the latter inducing overall higher antibody responses and pro-inflammatory cytokines.

## 5. Conclusions

In summary, we have developed TADV for avian influenza in poultry by utilising the specificity and high affinity binding properties of antibodies targeting chicken APCs. The scFv antibodies were chosen over the whole antibody for antigen targeting since, the former lacks Fc domain and reduces unspecific uptake of antigen. Here, H9HA antigen of AIV was recombinantly fused to scFv antibodies specific to chicken Dec205 and CD11c receptors. Both approaches using Dec205 and CD11c targeting led to higher levels of HI, virus MNT, and anti-HA antibodies compared to the untargeted H9HA antigen. However, CD11c targeting was better at eliciting an overall higher antibody response in vivo and stimulating chicken splenocytes to produce pro-inflammatory cytokines in vitro. These findings suggest that similar approaches could be used for the development of effective subunit vaccines for other poultry and livestock diseases.

## Data Availability

Data that support the findings of this study is included in the article and Supplementary Information.

## Supplementary Materials

The following are available online at https://www.mdpi.com/article/10.3390/vaccines9070784/s1. Figure S1: Schematic representation of single chain fragment variable antibody (scFv) and H9HA ectdomain fused scFv antibody expression cassettes; Table S1: Sequences of probes and primers used for qRT-PCR.
