# Supplementary material for: Targeting Haemagglutinin Antigen of Avian Influenza Virus to Chicken Immune Cell Receptors Dec205 and CD11c Induces Differential Immune-Potentiating Responses
Source: Vaccines (Basel). 2021 Jul 13;9(7):784. doi: 10.3390/vaccines9070784 (PMC8310205; doi:10.3390/vaccines9070784)
Supplement: Supplementary file 1 [file vaccines-09-00784-s001.zip › vaccines-1268838-supplementary.pdf]

Supplementary Materials:

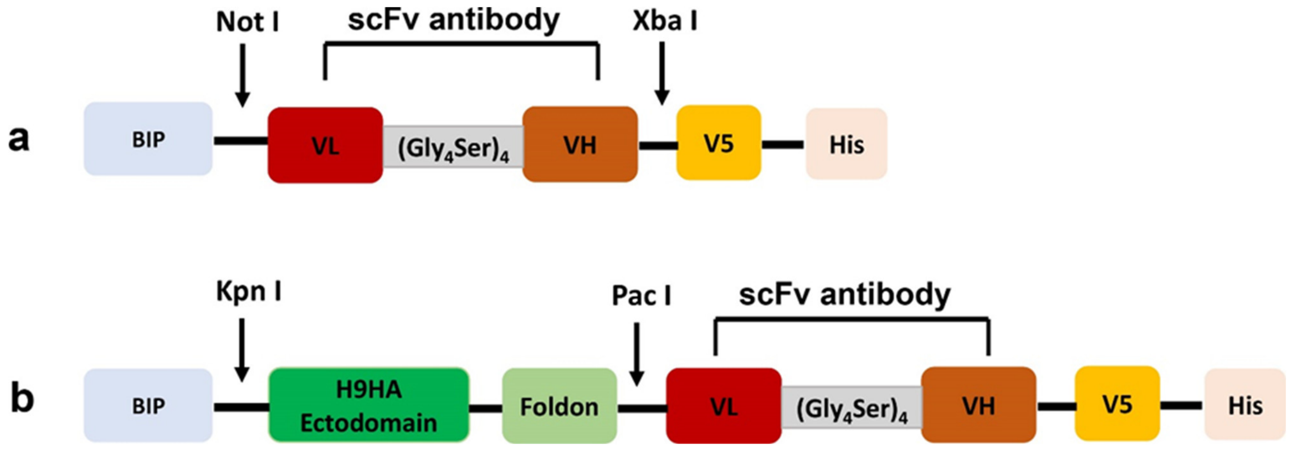

Figure S1. Schematic representation of single chain fragment variable antibody (scFv) and H9HA ectodomain fused scFv antibody expression cassettes **(a)** scFv construct. The expression cassette includes *Drosophila melanogaster* immunoglobulin heavy chain binding protein (BIP) secretion signal sequence followed by scFv antibody: variable light chain (vL), linker peptide $(Gly_4Ser)_4$ and variable heavy chain (vH). The V5 tag is for detection and His tag for purification **(b)** H9HA Foldon-scFv construct. The expression cassette includes BIP secretion signal sequence followed by H9HA ectodomain fused with trimerisation signal (indicated as foldon), conjugated to scFv antibody: variable light chain (vL), linker peptide $(Gly_4Ser)_4$ and variable heavy chain (vH).

Table S1. Sequences of probes and primers used for qRT-PCR.

Gene	Primer sequences (5'-3')	Probe sequence (5'-3')
IFNγ	**F**: GTGAAGAAGGTGAAAGA-TATCATGGA	**FAM**-TGGCCAA-GCTCCCGATGAACGA-**TAMRA**
	**R**: GCTTTGCGCTGGATTCTCA	
IL6	**F**: AACATGCGTCAGCTCCTGAAT	**FAM**- AGCAGCAC-CTCCCTCAAGGCACC-**TAMRA**
	**R:** TCTGCTAGGAACTTCTCCATT-GAA	
IL1β	**F:** GCTC-TACATGTCGTGTGTGATGAG	**FAM**-CCACACTGCAGCTGGAG-GAAGCC-**TAMRA**
	**R:** TGTCGATGTCCCGCATGA	
IL4	**F:** AACATGCGTCAGCTCCTGAAT	**FAM**-AGCAGCACC TCCCTCAAGGCACC-**TAMRA**
	**R**: TCTGCTAGGAACTTCTCCATT-GAA	
	**R:** TGGCACCGCAGCTCATT	
RPLPO-1	**F**: TTGGGCATCACCACAAAGATT	**FAM**-CATCAC-TCAGAATTTCAATGGTCCCTCGGG-**TAMRA**
	R: CCCACTTGTCTCCGGTCTTAA	

IFN: Interferon **IL**: Interleukin **RPLPO-1**: Ribosomal phosphoprotein lateral stalk subunit PO **F:** Forward R: Reverse FAM: 6-carboxyfluorescein **TAMRA**: Tetramethylrhodamine.
